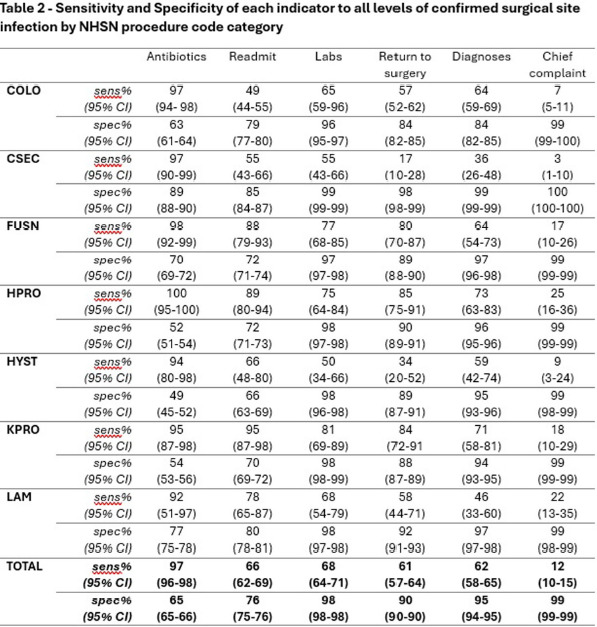# Epidemiologic and economic evaluation of automated surgical site infection indicators in a commercial surveillance software product

**DOI:** 10.1017/ash.2025.416

**Published:** 2025-09-24

**Authors:** Patrick Burke, Wanda Mullins, Thomas Fraser

**Affiliations:** 1 Cleveland Clinic; 2 Cleveland Clinic; 3 Cleveland Clinic Foundation

## Abstract

**Background:** The gold standard for surgical site infection (SSI) surveillance is 100% chart review, a practice neither efficient nor pragmatic for most large hospital systems.

Modern infection surveillance software uses indicators – specific data elements within the patient medical record - to report possible SSI for confirmation by infection preventionists (IPs). Using all available indicators has been shown to increase identification of SSIs and may approximate the gold standard but has been called “noisy” for including many patients with no SSI and costing precious surveillance time. Here, we describe our experience with evaluating the performance of our surveillance system. **Methods:** The setting for this study was the 21-hospital Cleveland Clinic health system with a uniform SSI surveillance plan and shared medical record. Our software, Bugsy (Epic Systems Corporation), employs six indicators for possible SSI: hospital readmission, return to surgery, positive microbiology tests, and chief complaint, physician diagnoses (billing codes), or administration of post-prophylaxis antibiotics suggestive of SSI. We extracted all possible SSIs, indicators, and confirmed infections for seven NHSN procedure code categories. We calculated the sensitivity and specificity of each indicator individually using OpenEpi v3.01 and estimated the cost associated with IP time spent on indicators that do not result in confirmed SSI. **Results:** From January to December 2023, 12,739 possible SSIs were reported with any indicator out of 26,276 inpatient procedures (48%). The frequency of procedures flagged for review ranged from 25% for CSEC to 78% for HYST. The number of procedures, possible SSI, and confirmed SSI with rates are shown in Table 1. The sensitivity and specificity of each indicator are shown in Table 2. Infection preventionists spent an average of 2 minutes reviewing each of 12,027 patient charts (401 hours) and determined there was no SSI, costing an estimated $18,602 annually. **Conclusion:** Nearly 50% of surgical patients were flagged for review for possible SSI with any indicator. Post-prophylaxis antibiotic was the most sensitive (97%) but least specific (65%) indicator. There was variability in indicator performance between procedure types. Readmission to the hospital was more sensitive in procedures with implants, e.g. KPRO and HPRO, than in procedures without, such as COLO and HYST. Evaluating the performance of possible SSI indicators enables IP programs to make data-driven and pragmatic decisions related SSI case finding practices. Tuning the indicator criteria within the software build may be necessary for optimization and presents an opportunity for IP time and cost savings.

**Figure:**
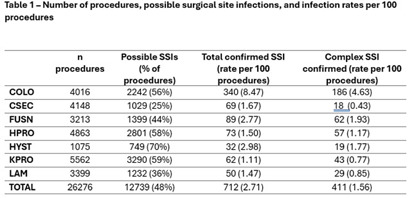

**Figure:**